# Impact of various extraction methods on fatty acid profile, physicochemical properties, and nutritional quality index of Pangus fish oil

**DOI:** 10.1002/fsn3.3431

**Published:** 2023-05-17

**Authors:** Nahidur Rahman, Shaharior Hashem, Shireen Akther, Jakia Sultana Jothi

**Affiliations:** ^1^ Department of Food Processing and Engineering Chattogram Veterinary and Animal Sciences University (CVASU) Chattogram Bangladesh; ^2^ Department of Aquaculture Bangladesh Agricultural University Mymensingh Bangladesh; ^3^ Laboratory of Marine Biology, Faculty of Agriculture Kyushu University Fukuoka Japan; ^4^ Laboratory of Postharvest Science, Faculty of Agriculture Kyushu University Fukuoka Japan

**Keywords:** acid silage, microwave‐assisted extraction, nutritional quality index, oxidative stability, Pangus fish oil, poly unsaturated fatty acid, soxhlet extraction, wet rendering

## Abstract

Marine fish are high in essential omega‐3 fatty acids, which are important for human health. This study evaluated the effects of four extraction methods (soxhlet extraction, SE; wet rendering, WR; acid silage, AS; microwave‐assisted extraction, MAE) on the oil yield, physicochemical properties, fatty acid profile, and nutritional quality index (NQI) of pangus fish oil. The oil yield ranged from 13.50% to 21.80%, with MAE having the highest yield. Furthermore, MAE oil has the lowest free fatty acid (0.70%), peroxides (2.08 Meq/kg), and saponification (287.27 mg/g KOH) value. There were no significant differences (*p* > .05) in the refractive index and melting point of oils among extraction techniques. A total of 25 fatty acids were identified. However, the maximum PUFA, MUFA, and SFA recovery was observed in the SE (19.15 mg/100 g), MAE (7.99 mg/100 g), and AS (17.33 mg/100 g), respectively. In terms of NQI, SE had higher PUFA/SFA, HH, and LA/ALA ratios, while AS had higher EPA + DHA, n‐3/n‐6, AI, TI, and FLQ indices. Furthermore, the MAE approach yielded better ratios of n‐3/n‐6 and HPI index, whereas the WR method yielded a higher AI index. Therefore, MAE would be the most efficient method for extracting pangus fish oil by considering both technical feasibility and quality indices including extraction yield, best physical properties, oxidative stability, and fatty acid contents.

## Graphical abstract

Since different manufacturers employ different extraction methods without the necessary knowledge to produce highest quality consumable fish oil. In order to increase the industrial use of these omega 3 enriched fish oils, high‐yield extraction methods that do not compromise the quality of the derived fish oils are required. In addition, as part of more sustainable production, safer ecological and energy‐saving methods to extract quality fish oil is a major challenge to the producer. It is therefore very important to determine the best extraction method by comparing physicochemical properties, fatty acid profile, and nutritional quality index (NQI) of pangus fish oil. In this study, microwave‐assisted extraction proved to be the most effective approach for recovering oil from pangus fish. The oil produced using this approach had the superior physical qualities, oxidative stability (AV, PV, FFV), included important fatty acids (higher content of MUFAs, EPA, and DPA and lower content of SFAs), and had the optimum nutritional quality indices (NQI). To the best of our knowledge, this is the first investigation into how different extraction methods affect pangus fish oil quality, extraction effectiveness, and fatty acid profiling.

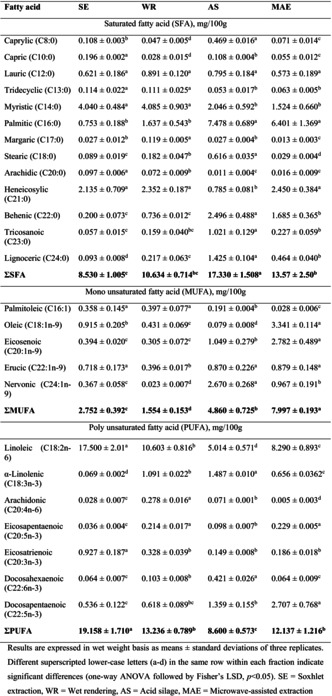

## 1 INTRODUCTION

Fish and fishery products are the most important sources of essential nutrients required for human consumption. Apart from food diversity, fisheries products play an important role in ensuring animal protein consumption through exportation from developing countries, one of which is native catfish (*Pangasius pangasius*), also locally known as pangus (Lestari & Purnamayati, 2020). Bangladesh is the world's second largest producer of pangus fish and the industry has the potential to be a dynamic sector of generating economic earnings and alleviating poverty (Hoque et al., 2021). In Bangladesh, pangus has become the single most important fish species, contributing a total production of 402,298 metric tons (MT) in 2020–2021 (DoF, 2022) with an 8.71% share of the total inland and marine fisheries. Abundance of this fish species is widespread in Bangladesh, particularly in fresh and brackish water, large rivers, food plains, estuaries, and canals.

However, people nowadays have become more health conscious and thereby looking for a healthier diet. Over the past two decades, polyunsaturated fatty acids (PUFAs) have gained much attention among scientists for their therapeutic and nutritional properties. Fish oil is now recognized as the prime source of these PUFAs. Today fish oil is highly valued for its positive role in human health and nutrition, owing to the presence of long‐chain n‐3 PUFAs, such as docosahexaenoic acid and eicosapentaenoic acid (Anandganesh et al., 2016; Hegde et al., 2016). Historically, fish oil has been studied for its significant role in human health, and hence, there has been an increasing demand for fish oil in food and pharmaceutical industries. Fish oil could be supplemented directly to food products to cover the fishy smell. Some previous studies also reported that fish oil can be used as a food additive in dairy products, butter, and baked goods (Pike & Jackson, 2010; Santhanam et al., 2015; Subroto et al., 2018; Zhong et al., 2018). Moreover, encapsulated fish oil was successfully incorporated in bread and bakery products (shortbread cookie and dark chocolates), meat and poultry products (chicken nuggets), as well as dairy products to improve their handling, storage, and oxidative stability (Jamshidi et al., 2019; Pourashouri et al., 2021; Umesha et al., 2015). The huge industry of fish processing accommodates diverse extraction and production of health‐promoting fish oil that can benefit both small fish oil processors and entrepreneurs. However, the crude fish oil contains variety of impurities and thus requires further extraction and purification to achieve quality characteristics suitable for human consumption (Crexi et al., 2010). Thus, rapid and reliable methods for the quantitative extraction of lipids from aquatic products are very important to preserve their nutrition and quality.

Fish oil can be extracted using a variety of methods, including dry rendering, wet rendering, acid hydrolysis, chemical extraction, mechanical pressing, and centrifugal force. Dry rendering involves hydraulic pressing followed by a cooker dryer and therefore requires a higher operation cost. Furthermore, the reaction between crude oil and the heated metal surface might degrade the color and result in inferior quality oil (Hicks & Verbeek, 2016). In contrast, wet rendering yields oil that has a neutral flavor, lighter color, and higher smoke point and hence is popular for fish body oil extraction (Suresh et al., 2019). Wet rendering and microwave‐assisted extraction are the two extraction methods that do not require the use of chemicals during the extraction process. Wet rendering involves steaming of fish muscle, which damages its cellular structure and extracts additional oil from the cooked fish (Nazir et al., 2017). This method, however, causes oxidation and degradation of heat‐labile substances in crude oil and thereby requires subsequent refining steps. Other oil extraction methods such as acid silage and soxhlet extraction involve separating a substance from its mixture by dividing a solute between two immiscible solvents. Addition of acids prior to lipid extraction in acid silage method could be very aggressive, and the extracts would be chemically degraded and unsuitable for fatty acid profiling. The soxhlet extraction method also involves the use of large amount of hazardous solvents. In addition, fish oils could be oxidized at the relatively high temperature as it takes longer time for complete extraction. Consequently, as part of more sustainable production, safer ecological and energy‐saving methods have been investigated for fish oil extraction. Among the different emerging green extraction techniques, microwave‐assisted extraction is gaining interest to obtain high‐quality fish oil. This method reduces energy consumption and also favors safer, robust, and controlled processes (Ghaly et al., 2013; Ozogul et al., 2018; Patil et al., 2012).

Furthermore, oil extraction is affected by several factors including extraction method, temperature, preliminary treatment, and contact time of the material with the solvent (Ghazali & Yasin, 2016). As a result, extraction procedures that result in high yields without compromising the quality of the extracted fish oils are required to increase the industrial application and utilization of these fats and oils of marine origin. Although several studies on nutritional content of pangus fish have been carried out by several researchers (Lestari & Purnamayati, 2020; Sugata et al., 2019), research on the effect of different extraction methods on the quality of fish oil is yet to be done. Therefore, this study aimed to determine the best extraction method by comparing physicochemical properties, fatty acid profile, and nutritional quality index (NQI) of pangus fish oil obtained from different extraction methods such as soxhlet extraction (SE), wet rendering (WR), acid silage (AS), and microwave‐assisted extraction (MAE).

## 2 MATERIALS AND METHODS

### 2.1 Chemicals

Ethanol, potassium iodide, sodium thiosulfate, acetic acid, hexane, di‐ethyl ether, potassium hydroxide, and chloroform were purchased from Sigma‐Aldrich (St. Louis, Missouri, USA) and were of analytical grade.

### 2.2 Sample preparation

Freshly captured experimental native pangus (*Pangasius pangasius*) fishes were sorted and identified. Fishes (average weight: 0.6 ± 0.15 kg, length: 38 ± 2 cm) were obtained from the fishermen of the main port of Chattogram, Bangladesh. Collected fishes were beheaded, eviscerated, washed, and immediately transported to the laboratory in ice boxes [2:1 (w/ w), ice to fish]. Muscles from fish belly flaps were cut into very small pieces (1–8 mm in diameter) and stored in airtight polythene bags at refrigerated temperatures (−20°C) for oil extraction and further analysis.

### 2.3 Oil extraction methods

#### 2.3.1 Soxhlet extraction (SE)

Soxhlet extraction process was carried out according to the standard AOAC method (AOAC, 2005). Fish samples (10 g) were weighed into cotton‐coated porous thimble in an electric balance (model: EK600i, Korea), which were then placed into the central chamber of the soxhlet apparatus (model: SER 148/3, Velp, Italy). A 250‐mL clean, oven‐dried, round‐bottomed flask was weighed and then connected to the soxhlet siphon and condenser; 80 mL of diethyl ether (40–60°C) was added to the flask and refluxed for 3 h. The heating flow rate was maintained low enough to prevent the solvent escaping from top of the condenser during refluxing. The solvent was then distilled off and the crude fish oil was collected, packaged in airtight PET bottle, and stored at −20°C.

#### 2.3.2 Wet rendering

Wet rendering technique was carried out according to the method described by Rubio‐Rodríguez et al. (2012) with slight modifications. Fish sample (100 g) was mixed with 150 mL of water in a 1000‐mL glass bottle and steamed at 105°C for 30 min. During the cooking process, sample was stirred in every 15 min. After cooking, the samples were transferred to a cloth bag and pressed manually. Obtained liquid was filtrated off by using separatory funnel. Ultimately, the oil phase was centrifuged at 15,344 *g* for 10 min and the crude fish oil was skimmed off, collected, and packaged in airtight PET bottle and further stored at −20°C.

#### 2.3.3 Acid silage

Acid silage technique was carried out according to the method described by Nazir et al. (2017). 3% of formic acid was added into minced samples [2:1 (w/v)] for acid silage preparation and kept at room temperature for 4–7 days. Produced liquid and cake were separated by filtration followed by centrifugation at 15,344 *g* for 10 min. Residual cake was pressed again to produce oil–water mixture and re‐centrifuged at 15,344 *g* for 10 min and the crude fish oil was collected, packaged in airtight PET bottle, and stored at −20°C.

#### 2.3.4 Microwave‐assisted extraction

Microwave‐assisted extraction (MAE) process was carried out according to the method described by Moreno et al. (2003). Fish muscles were spread on the rotary plate of a microwave oven (model: ME21K7010DS/AA, Samsung, South Korea). It was then heated at a high power level (600 W, 2450 MHz for 3–4 min). After that, the plate was removed from the oven and the oil was extracted by squeezing followed by pressing manually through a cloth mesh and filtration. Extracted oil was collected, packaged in airtight PET bottle, and stored at −20°C.

### 2.4 Yield determination

Crude fish oil fractions from three consecutive replicates were pooled together, and the yield was calculated as the percentage of oil extracted from the fish muscle. Yield was calculated using the following equation as described by Nazir et al. (2017).

$$\text{Yield}\;\left(\%\right):\frac{\text{Extracted fish oil}\;\left(g\right)}{\text{Weight of sample taken}\;\left(g\right)}\times 100.$$

### 2.5 Determination of physical properties of the extracted fish oil

Determination of melting point was carried out by the method described by Ndidiamaka and Ifeanyi (2018). Density, viscosity, and refractive index were also determined according to the recommended AOCS methods (AOCS, 1997). Density bottle and refractometer (model: R9500, Reed Instruments, China) were used to determine density and refractive index at 30°C, while viscosity of the oils was measured using a viscometer (model: DVII‐Brookfield, Middleboro, USA) with a small sample adapter (spindle‐62).

### 2.6 Determination of chemical properties of the extracted fish oils

Chemical properties of extracted pangus fish oils were monitored through measuring free fatty acids (FFA), acid value (AV), peroxide value (PV), iodine value (IV), saponification value (SV), saponification equivalent (SEq.), and ester value (EV). FFA was calculated as the percent of oleic acid by titration with 0.25 N NaOH solution. AV (mg KOH/g) was also determined by multiplying FFA (%) with 1.99, according to AOCS Official method, Ca 5a‐40 (AOCS, 1997). The PV was calculated as miliequivalents of peroxide/1000 g of oil by titrating against 0.1 N sodium thiosulphate solution along with a blank titration (AOAC, 2005). IV was determined according to the recommended method (AOAC, 2002) and expressed as g/100 g. The presence of soap or SV was determined as mg/g KOH by titration with a 0.5 N HCl solution (AOCS, 1997). Once the SV and AV have been determined, the difference between these two values represents the EV. Subsequently, SEq. was also determined using the following equation as described by Rahman et al. (2018).

$$\text{Saponification equivalent}\;\left(\mathrm{SEq}.\right)=\frac{56100}{\text{Saponification value}\;\left(\mathrm{SV}\right)\;\text{of the lipid}}$$

### 2.7 Determination of fatty acid profile of the extracted fish oils

For determining fatty acid profile, extracted pangus fish oils were subjected to methylation. Fatty acid methyl esters (FAMEs) of total lipid were prepared for gas chromatography–mass spectrometry (GC–MS) (model: GC‐2010 Plus, Shimadzu, Japan) analysis according to the method described by Harynuk et al. (2006). A quantity of 250 mg of oven‐heated (70–80°C) extracted lipids was taken in a test tube and saponified with methanolic sodium hydroxide solution (1.5 mL). The solution was heated at a sonicator for about 5 min. Two ml of boron trifluoride (BF_3_) was also added to the oil solution. Then, 5 mL of saturated sodium chloride (NaCl) and 1 mL of iso‐octane were also added to the test tube. The mixture was homogenized with vigorous shaking and allowed for 10 min to separate the clear‐colored FAME solution from a cloudy aqueous layer. Lastly, 1 mL of the organic layer on top was carefully pipetted off and inserted into a vial for GC–MS analysis. However, the fatty acids present in oil samples were measured in GC–MS using a MS detector at a predetermined wavelength. Prior to sample injection, hexane was injected three times to rinse GC–MS machine. A 1 μL sample from the vial containing 1 mL FAMEs solution is injected into GC–MS using a capillary column with CP‐Sil 5CB stationary phase with a preprogrammed oven temperature of 60–220°C with a temperature rise rate of 10°C/min. The carrier gas is 12 kPa pressurized Helium with a total rate of 11 mL/min and a split ratio of 1:50. From the chromatogram, qualitative and quantitative assessments of fatty acids including SFAs, MUFAs, and PUFAs were measured and identified (Nazir et al., 2017).

### 2.8 Determination of nutritional quality index (NQI)

Nutritional quality index (NQI) of pangus fish oils derived from different extraction methods was calculated as follows (Chen & Liu, 2020):

$$\text{Polyunsaturated fatty acid}/\text{saturated fatty acid ratio}\;\left(\text{PUFA}/\mathrm{SFA}\right):\frac{\text{∑PUFA}}{\text{∑SFA}}$$

$$\text{Index of atherogenicity}\;\left(\mathrm{AI}\right):\frac{\left[C12:0+\left(4\times C14:0\right)+C16:0\right]}{\text{∑UFA}}$$

$$\begin{array}{c} \text{Index of thrombogenicity}\;\left(\mathrm{TI}\right):\\ \frac{\left(C14:0+C16:0+C18:0\right)\;}{\left[\left(0.5\times \text{∑MUFA}\right)+\left(0.5\times \mathrm{\sum n}-6\;\text{PUFA}\right)+\left(3\times \mathrm{\sum n}-3\;\text{PUFA}\right)+\left(n-3/n-6\right)\right]} \end{array}$$

$$\text{Hypo}-\text{hypercholesterolemic ratio}\;\left(\mathrm{HI}\right):\frac{\left(\mathrm{cis}-C18:1+\text{∑PUFA}\right)}{\left(C12:0+C14:0+C16:0\right)}$$

$$\text{Health}-\text{promoting index}\;\left(\mathrm{HPI}\right):\frac{\text{∑UFA}}{\left[C12:0+\left(4\times C14:0\right)+C16:0\right]}$$

$$\mathrm{Sum}\;\text{of}\;\mathrm{EPA}\;\text{and}\;\mathrm{DHA}\;\left(\mathrm{EPA}+\mathrm{DHA}\right):C22:6\;n-3+C20:5\;n-3$$

$$\text{Fish lipid quality}\;\left(\mathrm{FLQ}\right):\frac{100\times \left(C22:6\;n-3+C20:5\;n-3\right)}{\mathrm{\sum FA}}$$

$$\text{Linoleic acid}/\alpha -\text{linolenic acid ratio}\;\left(\mathrm{LA}/\mathrm{ALA}\right):\frac{C18:2\;n-6}{C18:3\;n-3}$$

### 2.9 Statistical analysis

Each analysis was carried out in triplicates. Obtained data were stored in Microsoft Excel 2010 and the significant differences were determined by one‐way analysis of variance (ANOVA) followed by Fisher's LSD test using Minitab Statistical Software (Version: 19.1.1 0; Minitab, Ltd. United Kingdom). The significance level was measured at the level of *p* < .05.

## 3 RESULTS AND DISCUSSION

### 3.1 Effect of different extraction methods on the yield of pangus fish oil

The result of the yield percentage with different extraction methods is presented in Table 1. Significant differences were observed among the extraction methods (*p* < .05). However, the highest yield was reported in MAE (21.800 ± 0.233%) followed by WR (19.247 ± 0.661%), SE (13.503 ± 0.048%) and AS (10.233 ± 0.352%), respectively. However, oil yields from different extraction methods vary depending on whether the fish is cooked prior to extraction, the contact temperature, and whether certain solvents are used (Aryee & Simpson, 2009; Chantachum et al., 2000). The highest yield from the MAE method is attributed to the coagulation of protein, which releases both bound water and oil. The oil is further separated by pressing, resulting in an enhanced extraction (Taghvaei et al., 2014). The WR method also involves the coagulation of fish protein, so oil and solid materials get separated and skimmed off (Chantachum et al., 2000). Heating induced by both MAE and WR methods on muscle protein causes the formation of cross‐linking among the protein molecular side chains or intermolecular spaces, which might results to protein coagulation (Cabanillas & Novak, 2019). In contrast, the lower extraction efficiency in SE method might be attributed to the higher internal mass transfer resistance after initial recovery of most accessible oils, which might have slowed down the extraction rate (Rubio‐Rodríguez et al., 2012). Meanwhile, the AS method gives the lowest yield, because some of the fat remained emulsified as a stable skim fraction due to the action of acids or natural enzymes that cause the fats to bind tightly within the protein matrix (Taati et al., 2018). However, obtained results are in consistent with the findings of Afolabi et al. (2018) and Nazir et al. (2017) on the yield of oils from tuna's (*Thunnus albacares*) head and eel (*Monopterus albus*) fish. Therefore, MAE might be the most efficient method to recover crude oils from fish muscles compared to the other three methods.

**TABLE 1:** Yield of oils from different extraction methods.

Extraction methods	Yield (%)
SE	13.503 ± 0.048^c^
WR	19.247 ± 0.661^b^
AS	10.233 ± 0.352^d^
MAE	21.800 ± 0.233^a^

*Note*: Results are expressed in wet weight basis as means ± standard deviations of three replicates. Different superscripted lower‐case letters (a–d) in the same column within each fraction indicate significant differences (*p* < .05).

Abbreviations: AS, Acid silage; MAE, Microwave‐assisted extraction; SE, Soxhlet extraction; WR, Wet rendering.

### 3.2 Effect of different extraction methods on the physical properties of pangus fish oil

Physical properties including density, refractive index, viscosity, and melting point of pangus fish oils obtained from four different extraction methods were investigated and their subsequent effect is given in Table 2. The viscosity ranged from 43.00 ± 0.50 to 52.00 ± 0.40 cP with significant differences among the extraction methods (*p* < .05). Furthermore, refractive index, density, and melting points also ranged from 1.455 ± 0.003 to 1.460 ± 0.002, 0.909 ± 0.001 to 0.913 ± 0.001 g/mL, and 32.50 ± 0.50 to 34.23 ± 0.25°C, respectively, with nonsignificant differences observed in density values (*p* > .05).

**TABLE 2:** Physical properties of oils from different extraction methods.

Physical properties	SE	WR	AS	MAE
Refractive index	1.455 ± 0.003^a^	1.460 ± 0.002^a^	1.459 ± 0.002^a^	1.457 ± 0.004^a^
Density (g/mL)	0.909 ± 0.002^bc^	0.912 ± 0.001^ab^	0.913 ± 0.001^a^	0.909 ± 0.001^c^
Melting point (°C)	32.50 ± 0.50^b^	34.23 ± 0.25^a^	33.47 ± 0.50^a^	33.50 ± 0.50^a^
Viscosity (cP)	46.33 ± 1.33^c^	48.00 ± 0.50^b^	52.00 ± 0.40^a^	43.00 ± 0.50^d^

*Note*: Results are expressed in wet weight basis as means ± standard deviations of three replicates. Different superscripted lower‐case letters (a–d) in the same row within each fraction indicate significant differences (*p* < .05).

Abbreviations: AS, Acid silage; MAE, Microwave‐assisted extraction; SE, Soxhlet extraction; WR, Wet rendering.

The refractive index of an oil or fat is somewhat dependent on its degree of unsaturation, and a higher refractive index indicates the presence of more unsaturated materials. It also measures the changes in unsaturation due to different extraction methods. The refractive index of oils varies according to molecular weight, chain length of fatty acids, degree of unsaturation, and degree of conjugation (Andhale et al., 2017). However, obtained values of refractive index are within the standard limits (1.40–1.47) as recommended for edible oils by Abdulkadir et al. (2010).

Density is another important factor that influences oil absorption capacity and mass transfer rate owing to different extraction methods. The density of fish oil varies depending on the degree of heat treatment used to extract the oils. Since oil occupies more volume due to molecular diffusion caused by heat treatment, MAE followed by SE resulted in slightly lower density values in this study. However, regardless of extraction method, density values are lower than water (1.000 g/mL) and compatible with other edible oils such as canola oil (0.913 g/mL) and olive oil (0.908 g/mL), according to Sahasrabudhe et al. (2017).

The physical properties of oils and fats, such as hardness and thermal behaviors, are described by melting point. The extracted fish oils had melting points lower than cottonseed oil (42.8°C), sheep tallow (42°C), and palm oil (35°C), but higher than sunflower oil (−17°C), soybean oil (−16°C), and olive oil (−6°C) (Engineering toolbox, 2008). The melting point, on the other hand, is affected by the degree of unsaturation and chain length. However, the SE method reports a significantly lower melting point due to a lower level of saturation, which is consistent with the results published by Lestari and Purnamayati (2020).

Another important factor influencing the physical properties of fish oil is its viscosity. Impurities such as free fatty acids, proteins, pigments, moisture, volatile compounds, and the degree of unsaturation can all have an impact on the viscosity of oils or fats (Suseno et al., 2015). In this study, the viscosity of microwave‐assisted fish oil was significantly lower than that of SE, WR, and AS methods. The MAE method employs microwave energy at high temperatures to reduce intermolecular attractions between molecules, lowering density and making the oil less viscous. However, the resulting fish oils have the same viscosity as sardine oil (51.70 cP), as reported by Suseno et al. (2015). Higher viscosity, on the other hand, generally indicates lower purity of oil. Meanwhile, the selectivity of the AS method might be poor, resulting in the extraction of all classes of lipids and other molecules, which may be the cause of the increased viscosity in the resulting oil. However, highly viscous oils or fats require additional refining to lower their viscosity.

### 3.3 Effect of different extraction methods on the chemical properties of pangus fish oil

The values of free fatty acid (FFA), acid value (AV), peroxide value (PV), iodine value (IV), saponification value (SV), saponification equivalent (SEq.), and ester value (EV) of pangus fish oil obtained by different extraction methods are presented in Table 3. Based on differentiating the extraction methods, AV, FFA, SV, SEq., IV, PV, and EV of the extracted fish oils varied from 1.402 ± 0.008 to 1.523 ± 0.008 (mg KOH/g), 0.704 ± 0.004 to 0.766 ± 0.004 (%, as oleic acid), 162.964 ± 0.004 to 195.286 ± 0.654 (mg/g KOH), 287.272 ± 0.964 to 344.248 ± 0.008, 49.345 ± 0.514 to 61.187 ± 0.539 (g/100 g), 2.081 ± 0.703 to 4.645 ± 0.482 (Meq/kg), and 161.495 ± 0.003 to 193.885 ± 0.647, respectively, and showed significant differences among the extraction methods (*p* < .05).

**TABLE 3:** Chemical properties of oils from different extraction methods.

Chemical properties	SE	WR	AS	MAE
AV (mg KOH/g)	1.469 ± 0.006^b^	1.523 ± 0.008^a^	1.442 ± 0.008^c^	1.402 ± 0.008^d^
FFA (% oleic acid)	0.738 ± 0.003^b^	0.766 ± 0.004^a^	0.725 ± 0.004^c^	0.704 ± 0.004^d^
SV (mg/g KOH)	162.964 ± 0.004^d^	186.697 ± 0.011^b^	177.555 ± 0.502^c^	195.286 ± 0.654^a^
SE	344.248 ± 0.008^a^	300.487 ± 0.017^c^	315.960 ± 0.894^b^	287.272 ± 0.964^d^
IV (g/100 g)	61.187 ± 0.539^a^	49.345 ± 0.514^d^	51.708 ± 0.365^c^	54.439 ± 0.591^b^
PV (Meq/kg)	4.146 ± 0.292^b^	3.308 ± 0.437^c^	4.645 ± 0.482^a^	2.081 ± 0.703^d^
EV	161.495 ± 0.003^d^	185.174 ± 0.005^b^	176.113 ± 0.508^c^	193.885 ± 0.647^a^

*Note*: Results are expressed in wet weight basis as means ± standard deviations of three replicates. Different superscripted lower‐case letters (a–d) in the same row within each fraction indicate significant differences (*p* < .05).

Abbreviations: AS, Acid silage; AV, Acid value; EV, Ester value; FFA, Free fatty acid; IV, Iodine value; MAE, Microwave‐assisted extraction; PV, Peroxide value; SE, Saponification equivalent; SE, Soxhlet extraction; SV, Saponification value; WR, Wet rendering.

The acid value is an important quality parameter of fats or oils related to the presence of free fatty acids and other nonlipid acidic compounds such as acetic acid. The acidity of crude oil depends on several factors, including the oil composition, extraction procedure, and the freshness of the raw materials (Rubio‐Rodríguez et al., 2012). Table 3 illustrated the highest acid value in WR (1.523 ± 0.008 mg KOH/g), followed by SE (1.469 ± 0.006 mg KOH/g), AS (1.442 ± 0.008 mg KOH/g), and MAE (1.402 ± 0.008 mg KOH/g), respectively. However, higher acidity in WR might be attributed to the hydrolysis of triglycerides during heating and the exposure of fish muscles to air. The lower acid value in MAE, on the other hand, could be attributed to the shortened extraction time caused by microwave heating. A lower acid value usually indicates purity and suitability of oils or fats, whereas a higher value is associated with rancidity. Furthermore, the acid value of edible oils should not exceed 4 mg KOH/g (Sasongko et al., 2017), and the results of this study are within the recommended limits.

Unsaturated fatty acids with double bonds in their structures react with heat, air, or water to form free fatty acids that affect the quality of fish oil (Nazir et al., 2017). FFA value usually indicates the degree of hydrolysis or oxidation. The value of standard free fatty acids in lipids for edible purposes is ≤1.5% (IFOS, 2014). However, FFA values in all the extracted fish oils are within the acceptable range, indicating no or less lipid degradation occurred during or after the extraction procedures (Table 3). Among the extracted fish oils, the highest FFA was measured in WR (0.766 ± 0.004%), followed by SE (0.738 ± 0.003%), AS (0.725 ± 0.004%), and MAE (0.704 ± 0.004%), respectively. However, the lower the FFA value, the better the oil quality. Aryee and Simpson (2009) also reported similar FFA content (0.6%–1.2%) in salmon oil. Furthermore, the lowest FFA in MAE might be attributed to the shortened extraction time. Accordingly, oil that was exposed to heat and air for a longer period of time had a higher FFA value (Chantachum et al., 2000).

Saponification is the process of breaking down a neutral fat into glycerol and fatty acids through alkaline conditioning. The saponification value measures the amount of fatty acids found in fish oil. A high saponification value indicates that the oil contains fatty acids with lower molecular weights (Low & Ng, 1987). In this study, the highest saponification value was observed in MAE (195.286 ± 0.654 mg/g KOH) followed by WR (186.697 ± 0.011 mg/g KOH), AS (177.555 ± 0.502 mg/g KOH), and SE (162.964 ± 0.004 mg/g KOH), respectively. Regardless of SE method, saponification values of all the extracted fish oils are within the standard limit (min. 170 mg/g KOH) as reported by Sasongko et al. (2017). The increased saponification value might be attributed to the oxidation and polymerization reactions induced by MAE during oil extraction (Jacobson et al., 2008), whereas the lower values of soxhlet extracted oils might be due to impurities being transferred to oil during solvent extraction (Özcan et al., 2020). However, obtained saponification values have close similarities with cottonseed oil (175 to 198 mg/g KOH) and castor oil (175 to 180 mg/g KOH) as described by Rahman et al. (2018).

Fats or oils usually contain unsaturated and some saturated fatty acids such as myristic acid, palmitic acid, as well as some unsaponifiable matter. In this study, the highest SEq. value was reported in SE (344.248 ± 0.008), followed by AS (315.960 ± 0.894), WR (300.487 ± 0.017), and MAE (287.272 ± 0.964), respectively. However, saponification equivalent is proportional to the average chain length of the fatty acids present in the respective fish oil (Islam et al., 2012), whereas saponification value demonstrates the reverse trend. The greater the saponification value, the shorter the average chain length of fatty acids and the lower the average molecular weight of triglycerides. Obtained results are in accordance with the result published by Rahman et al. (2018) who concluded that oils with higher acidity generally have saponification equivalents of around 290.80. In addition, higher saponification equivalent indicates the presence of significantly higher fatty acids. Thus, findings of saponification equivalent clearly indicate that soxhlet extracted pangus fish oil might contain considerable amount of saturated and unsaturated fatty acids compared to other extraction methods. Results of the present study regarding fatty acids also elucidated the similar trends for soxhlet extracted fish oil.

The amount of iodine absorbed measures the number of reactive double bonds or unsaturated bonds present in oils or fats. This value is used to determine the extent of oils to be oxidized. The results of this study indicate that iodine value in the SE (61.187 ± 0.539 g/100 g) is higher than that of MAE (54.439 ± 0.591 g/100 g), AS (51.708 ± 0.365 g/100 g), and WR (49.345 ± 0.514 g/100 g), respectively. However, a higher iodine value indicates higher unsaturation of fats and oils. Thus, higher iodine value in the oils from SE method indicates higher unsaturation and is prone to oxidation. In addition, iodine value also regulates the melting point of fish oil. According to Hasibuan (2012), higher the double‐bonded unsaturated fatty acids, more liquid would be the fish oil and vice versa. This trend was also followed by the melting point values in this study. However, obtained iodine values are higher than that of the oil from tilapia 9.13 g/100 g and lower than that of fresh mackerel 121.60 g/100 g (Ndidiamaka & Ifeanyi, 2018; Nugroho et al., 2014).

The peroxide value determines the extent to which the oil undergoes rancidity during processing, extraction, and storage. Besides, it also used to monitor the quality and stability of fats and oils. The smaller the peroxide value, the better the quality of the oil. In this study, fish oils obtained from AS (4.645 ± 0.482 Meq/kg) and SE (4.146 ± 0.292 Meq/kg) methods contained higher peroxides than WR (3.308 ± 0.437 Meq/kg) and MAE (2.081 ± 0.703 Meq/kg), respectively. Degradation of fish muscles due to prolonged cooking and exposure to air releases more free ions; thus, wet‐rendered oils contained more free ions, resulting in higher oxidation rate. The higher oxidation rate detected in AS extraction is also predicted by natural enzymes or protein denaturation caused by acids. Denaturation of the protein molecule weakens the unsaturated bonds or links, increasing the likelihood of oxidation. Gracey et al. (1999) reported that oil with a peroxide value of less than 5 Meq/kg can be considered fresh oil, while oil with a peroxide value of 7.5 Meq/kg is unacceptable for human consumption (Robards et al., 1988). It is known from this study that the peroxide values of all the extracted oil samples are relatively good as they are within the recommended limits. Similar results were also reported by Nazir et al. (2017).

Esters are naturally occurring components in fats and oils that are responsible for flavor development and pleasant odors. The ester value indicates the amount of alkali consumed to saponify the esters contained in fats or oils. The oils extracted using the MAE (193.885 ± 0.647) method represent higher ester values than the WR (185.174 ± 0.005), AS (176.113 ± 0.508) and SE (161.495 ± 0.003) methods, respectively. Because of its volumetric heating effects, microwave heating accelerates reaction rates and can directly penetrate the fish muscle to hydrolyze the esters, increasing the ester value in extracted oil (Abdulbari et al., 2011). In contrast, due to extended extraction time in other methods, volatile compounds such as esters might be vaporized and can be lost with water vapor, resulting in decreased ester vale (Cao, 2012). The results of this study, however, are slightly lower than the ester value of striped catfish (*Pangasius sutchi*) oil previously reported by Islam et al. (2012).

### 3.4 Effect of different extraction methods on fatty acid profile of pangus fish oil

Fatty acid composition of extracted pangus fish oils are depicted in Table 4. A total of 25 fatty acids comprising SFAs, MUFAs, and PUFAs were identified and the concentration was calculated based on retention time. Oil obtained from AS method had significantly higher levels of SFAs (17.330 ± 1.508 mg/100 g) compared to MAE (13.57 ± 2.50 mg/100 g), WR (10.634 ± 0.714 mg/100 g), and SE (8.530 ± 1.005 mg/100 g), respectively (*p* < .05). However, myristic acid was reported to be the major component of SFA in both SE (4.040 ± 0.484 mg/100 g) and WR (4.085 ± 0.903 mg/100 g) methods, while palmitic acid was found to be the major SFA in both AS (7.478 ± 0.689 mg/100 g) and MAE (6.401 ± 1.369 mg/100 g) methods. MUFAs were found to be the most abundant in pangus fish oils extracted using MAE (7.997 ± 0.193 mg/100 g) method followed by AS (4.860 ± 0.725 mg/100 g), SE (2.752 ± 0.392 mg/100 g), and WR (1.554 ± 0.153 mg/100 g), respectively. Meanwhile, pangus fish oil extracted using the SE (19.158 ± 1.710 mg/100 g) method contained the most PUFAs, followed by WR (13.236 ± 0.789 mg/100 g), MAE (12.137 ± 1.216 mg/100 g), and AS (8.600 ± 0.573 mg/100 g) methods, respectively. However, the lowest recovery of PUFAs in AS method might be attributed to the emulsion formation during oil extraction (Hajeb et al., 2015). In addition, gentle heating during extraction procedure prevents oxidation and can therefore be used to efficiently recover PUFAs. Previously, researchers reported EPA and DHA as the main PUFAs in marine fish (Ozogul et al., 2018). In this study, the MAE (0.229 ± 0.005 mg/100 g) and WR (0.214 ± 0.017 mg/100 g) methods yielded the highest EPA content, followed by AS (0.098 ± 0.007 mg/100 g) and SE (0.036 ± 0.004 mg/100 g) methods, respectively. However, similar findings were also reported by Hajeb et al. (2015) who concluded that higher amounts of EPA can be recovered by using the WR method. In addition, DHA contents in extracted fish oils ranged from 0.064 ± 0.007 to 0.421 ± 0.026 mg/100 g and did not show significant differences in both SE and MAE methods (*p* > .05). Therefore, the AS extraction method is ideal for recovering DHA, whereas the WR and MAE extraction methods are ideal for recovering EPA. Furthermore, MAE followed by AS methods are much better in extracting MUFAs. Previous study of Sathivel et al. (2003) also reported similar findings that contents of MUFAs (C16:1, C18:1) obtained from MAE method were higher compared to other methods. Moreover, AS method produced the highest SFAs content, while SE method produced the least count. The lowest SFAs in fish oils were also observed by Ozogul et al. (2018). However, the interactions between different extraction methods to recover fatty acids are in line with previous study undertaken by Aursand et al. (1994), where they have concluded that abundance of both polar and nonpolar fats within the fish muscle, degree of saturation, and using solvents or not during extraction might have caused the variations in fatty acid contents. Moreover, consumption of unsaturated fatty acids (UFAs) is more important than saturated fatty acids (SFAs) for health and wellbeing (Lawrence, 2010). Despite having the highest PUFA contents, SE requires a longer extraction time as well as high purity solvents. Furthermore, contamination with potentially hazardous and flammable organic solvents, as well as the emission of toxic compounds during the SE procedure, may be a cause for concern. In this context, MAE might be effective in maintaining health and preserving better nutritional quality.

**TABLE 4:** Fatty acid profiles of oils from different extraction methods.

Fatty acid	SE	WR	AS	MAE
*Saturated fatty acid (SFA), mg/100 g*				
Caprylic (C8:0)	0.108 ± 0.003^b^	0.047 ± 0.005^d^	0.469 ± 0.016^a^	0.071 ± 0.014^c^
Capric (C10:0)	0.196 ± 0.002^a^	0.028 ± 0.015^d^	0.108 ± 0.004^b^	0.055 ± 0.012^c^
Lauric (C12:0)	0.621 ± 0.186^a^	0.891 ± 0.120^a^	0.795 ± 0.184^a^	0.573 ± 0.189^a^
Tridecyclic (C13:0)	0.114 ± 0.022^a^	0.111 ± 0.025^a^	0.053 ± 0.017^b^	0.063 ± 0.005^b^
Myristic (C14:0)	4.040 ± 0.484^a^	4.085 ± 0.903^a^	2.046 ± 0.592^b^	1.524 ± 0.660^b^
Palmitic (C16:0)	0.753 ± 0.188^b^	1.637 ± 0.543^b^	7.478 ± 0.689^a^	6.401 ± 1.369^a^
Margaric (C17:0)	0.027 ± 0.012^b^	0.119 ± 0.005^a^	0.027 ± 0.004^b^	0.013 ± 0.003^c^
Stearic (C18:0)	0.089 ± 0.019^c^	0.182 ± 0.047^b^	0.616 ± 0.035^a^	0.029 ± 0.004^d^
Arachidic (C20:0)	0.097 ± 0.006^a^	0.072 ± 0.009^b^	0.011 ± 0.004^c^	0.016 ± 0.009^c^
Heneicosylic (C21:0)	2.135 ± 0.709^a^	2.352 ± 0.187^a^	0.785 ± 0.081^b^	2.450 ± 0.384^a^
Behenic (C22:0)	0.200 ± 0.073^c^	0.736 ± 0.012^c^	2.496 ± 0.488^a^	1.685 ± 0.365^b^
Tricosanoic (C23:0)	0.057 ± 0.015^c^	0.159 ± 0.040^bc^	1.021 ± 0.129^a^	0.227 ± 0.059^b^
Lignoceric (C24:0)	0.093 ± 0.008^d^	0.217 ± 0.063^c^	1.425 ± 0.104^a^	0.464 ± 0.040^b^
∑SFA	8.530 ± 1.005^c^	10.634 ± 0.714^bc^	17.330 ± 1.508^a^	13.57 ± 2.50^b^
*Mono unsaturated fatty acid (MUFA), mg/100 g*				
Palmitoleic (C16:1)	0.358 ± 0.145^a^	0.397 ± 0.077^a^	0.191 ± 0.004^b^	0.028 ± 0.006^c^
Oleic (C18:1n‐9)	0.915 ± 0.205^b^	0.431 ± 0.069^c^	0.079 ± 0.008^d^	3.341 ± 0.114^a^
Eicosenoic (C20:1n‐9)	0.394 ± 0.020^c^	0.305 ± 0.072^c^	1.049 ± 0.279^b^	2.782 ± 0.489^a^
Erucic (C22:1n‐9)	0.718 ± 0.173^a^	0.396 ± 0.017^b^	0.870 ± 0.226^a^	0.879 ± 0.148^a^
Nervonic (C24:1n‐9)	0.367 ± 0.058^c^	0.023 ± 0.007^d^	2.670 ± 0.268^a^	0.967 ± 0.191^b^
∑MUFA	2.752 ± 0.392^c^	1.554 ± 0.153^d^	4.860 ± 0.725^b^	7.997 ± 0.193^a^
*Poly unsaturated fatty acid (PUFA), mg/100 g*				
Linoleic (C18:2n‐6)	17.500 ± 2.01^a^	10.603 ± 0.816^b^	5.014 ± 0.571^d^	8.290 ± 0.893^c^
α‐Linolenic (C18:3n‐3)	0.069 ± 0.002^d^	1.091 ± 0.022^b^	1.487 ± 0.010^a^	0.656 ± 0.0362^c^
Arachidonic (C20:4n‐6)	0.028 ± 0.007^c^	0.278 ± 0.016^a^	0.071 ± 0.001^b^	0.005 ± 0.003^d^
Eicosapentaenoic (C20:5n‐3)	0.036 ± 0.004^c^	0.214 ± 0.017^a^	0.098 ± 0.007^b^	0.229 ± 0.005^a^
Eicosatrienoic (C20:3n‐3)	0.927 ± 0.187^a^	0.328 ± 0.039^b^	0.149 ± 0.008^b^	0.186 ± 0.018^b^
Docosahexaenoic (C22:6n‐3)	0.064 ± 0.007^c^	0.103 ± 0.008^b^	0.421 ± 0.026^a^	0.064 ± 0.009^c^
Docosapentaenoic (C22:5n‐3)	0.536 ± 0.122^c^	0.618 ± 0.089^bc^	1.359 ± 0.155^b^	2.707 ± 0.768^a^
∑PUFA	19.158 ± 1.710^a^	13.236 ± 0.789^b^	8.600 ± 0.573^c^	12.137 ± 1.216^b^

*Note*: Results are expressed in wet weight basis as means ± standard deviations of three replicates. Different superscripted lower‐case letters (a–d) in the same row within each fraction indicate significant differences (one‐way ANOVA followed by Fisher's LSD, *p* < .05).

Abbreviations: AS, Acid silage; MAE, Microwave‐assisted extraction; SE, Soxhlet extraction; WR, Wet rendering.

### 3.5 Effect of different extraction methods on the NQI of pangus fish oils

The nutritional value of dietary food ingredients is repeatedly evaluated through nutritional quality index (NQI). It is calculated by several indices of fatty acid composition, which provides greater insights regarding the possible health effects of certain fatty acids such as lauric acid (C12:0), myristic acid (C14:0), and palmitic acid (C16:0), which has been evidenced to increase the total serum cholesterol which eventually causes coronary heart diseases (Ulbricht & Southgate, 1991). However, the nutritional quality index (NQI) of pangus fish oil extracted by different extraction methods is summarized in Table 5. Significant differences were observed in PUFA/SFA and FLQ indices regardless of different extraction methods (*p* < .05). Significantly lower n‐3/n‐6 rations were observed in SE and WR methods, respectively (*p* < .05). Furthermore, AS method yielded better EPA + DHA, AI, TI, and FLQ indices, while better HA and LA/ALA rations were observed in SE method (Table 5).

**TABLE 5:** Nutritional quality index (NQI) of oils from different extraction methods.

NQI	SE	WR	AS	MAE
PUFA/SFA	2.252 ± 0.094^a^	1.248 ± 0.104^b^	0.498 ± 0.044^d^	0.905 ± 0.094^c^
EPA + DHA	0.099 ± 0.011^c^	0.317 ± 0.025^b^	0.519 ± 0.028^a^	0.293 ± 0.014^b^
n‐3/n‐6	0.010 ± 0.002^c^	0.129 ± 0.009^b^	0.398 ± 0.044^a^	0.398 ± 0.044^a^
AI	0.807 ± 0.065^b^	1.275 ± 0.201^a^	1.223 ± 0.156^a^	0.668 ± 0.123^b^
TI	0.459 ± 0.026^b^	0.559 ± 0.085^b^	0.892 ± 0.097^a^	0.511 ± 0.238^b^
HH	3.703 ± 0.182^a^	2.048 ± 0.188^b^	0.845 ± 0.076^c^	1.865 ± 0.302^b^
HPI	1.252 ± 0.088^ab^	0.799 ± 0.138^c^	0.827 ± 0.112^bc^	1.627 ± 0.411^a^
LA/ALA	250.6 ± 31.8^a^	9.717 ± 0.722^b^	3.370 ± 0.372^b^	12.69 ± 1.76^b^
FLQ	0.329 ± 0.066^d^	1.249 ± 0.129^b^	1.686 ± 0.049^a^	0.954 ± 0.093^c^

*Note*: Results are expressed in wet weight basis as means ± standard deviations of three replicates. Different superscripted lower‐case letters (a–d) in the same row within each fraction indicate significant differences (*p* < .05).

Abbreviations: AI, Index of atherogenicity; AS, Acid silage; FLQ, Fish lipid quality; HH, Hypo and Hypercholesterolemia ratio; HPI, Health promoting index; MAE, Microwave‐assisted extraction; SE, Soxhlet extraction; TI, Index of thrombogenicity; WR, Wet rendering.

The PUFA/SFA ratio is currently one of the main parameters to assess the nutritional quality of seafood and dietary fat. The highest PUFA/SFA ratio in the extracted fish oils was observed in SE (2.252 ± 0.094) followed by WR (1.248 ± 0.104), MAE (0.905 ± 0.094), and AS (0.498 ± 0.044) methods, respectively. However, PUFA/SFA ratio is recommended to be higher than 0.4, so as to reduce the risk of cardiovascular, autoimmune, and other chronic diseases (Simopoulos, 2002). Besides, a lower PUFA/SFA ratio indicates a higher level of dietary saturated fatty acids, which is considered as the major risk factors for cardiovascular disease. In the current study, this ratio has exceeded the minimum recommended limit for all the oils regardless of extraction methods. The greatest increase in PUFA/SFA ratio might be attributed to the maximal absorption of PUFAs while extracting the oils (Karimian‐Khosroshahi et al., 2016).

EPA and DHA are the long‐chain n‐3 fatty acids, which are the precursors of hormones known as eicosanoids and docosanoids that play important roles in biological processes in the body. A daily intake of approximately 500–1000 mg of EPA and DHA has been recommended by American Heart Association to reduce the risk of coronary heart diseases (Huynh & Kitts, 2009). Previously, Hosseini et al. (2014) reported that cooking procedure reduces the content of EPA + DHA in cooked kutum roach (*Rutilus frisii kutum*). However, present study reported that AS (0.519 ± 0.028) and SE (0.099 ± 0.011) methods showed significant differences in EPA + DHA content regardless of MAE (0.293 ± 0.014) and WR (0.317 ± 0.025) methods, respectively (*p* < .05). However, the greatest recovery of EPA + DHA content might be attributed to reduced physical losses during oil extraction from fish muscle.

The n‐3/n‐6 ratio is considered as the useful indicator for comparing relative nutritional values of fish oils. According to health recommendations, the n‐3/n‐6 ratio should be lower than 0.67 thereby to reduce the incidence of cardiovascular disease, pro‐inflammation, cancer, and obesity (Simopoulos, 2002). In addition, the lower n‐3/n‐6 ratio enables better utilization of n‐3 fatty acids in human body. Results from this study showed that all the extracted oils have a very good n‐3/n‐6 ratio with SE (0.010 ± 0.002) and WR (0.129 ± 0.009) having a lower ratio than MAE (0.398 ± 0.044) and AS (0.398 ± 0.044) methods, respectively. Solvent extracted oils had the lowest n‐3/n‐6 ratio because of the prompt absorption of linolenic acid and other n‐6 fatty acids.

Atherogenicity index (AI) and Thrombogenic index (TI) are the two indices proposed by Ulbricht and Southgate (1991), which characterize the atherogenic and thrombogenic potential of the fatty acids relative to other indices. Lower values of both indices indicate better nutritional value of fatty acids, so diets with lower AI and TI values may reduce the potential risk of coronary heart disease (Karimian‐Khosroshahi et al., 2016). Lower values are recommended for AI (<1) and TI (<1), indicating positive health benefits from the product (Krešić et al., 2019). In this study, higher AI indices were observed in both AS (1.223 ± 0.156) and WR (1.275 ± 0.201) methods and exceeded the expected range for fish oils. In this context, coconut oil was reported to be a highly atherogenic food with an AI value of 13.63. While raw mackerel, olive, and sunflower oil with AI values of 0.28, 0.14, and 0.07, respectively, have been reported to be the lower atherogenic foods (Chen et al., 2004). Meanwhile, oils obtained from MAE (0.511 ± 0.238), WR (0.559 ± 0.085), and SE (0.459 ± 0.026) methods had similar TI index except AS (0.892 ± 0.097). However, obtained TI values in the present study are within the expected range. Previously, raw mackerel oil was reported to be highly antithrombogenic with a TI value of 0.16, followed by sunflower oil (0.28) and olive oil (0.32) (de Alba et al., 2019).

The effect of specific fatty acids on cholesterol metabolism can be represented by hypo‐to‐hyper cholesterolamic ratio (HH). A higher HH ratio is desirable as it represents higher nutritional value. In this study, the highest HH value was reported in SE (3.703 ± 0.182) method followed by WR (2.048 ± 0.188), MAE (1.865 ± 0.302), and AS (0.845 ± 0.076) methods, respectively. In other studies, the HH ratio in fish and fishery products ranged in between 0.25 and 4.83 (Hosseini et al., 2014). MAE and WR methods showed nonsignificant effects on HH ratio (*p* > .05). In contrast, SE method significantly increased the HH ratio, while AS showed the least HH ratio (*p* < .05). However, HH ratio of all the extracted oils except for AS method was beyond the optimum value (HH > 1) as described by Krešić et al. (2019).

The health‐promoting index (HPI) was proposed by Chen et al. (2004) to evaluate the nutritional value of dietary fat focusing on the effect of fatty acid composition on cardio‐vascular diseases (CVD). It is believed that fats or oils with higher HPI values are more beneficial to human health. HPI is the inverse of IA. This study showed significant differences in HPI indices depending on the extraction methods (*p* < .05). However, as reported by Chen et al. (2004) and Bobe et al. (2007), the obtained values of the HPI indices are much larger than those of butter (0.37–0.66) and cheese (0.29–0.46).

FLQ was primarily used to determine fish lipid quality, which calculates the sum of EPA and DHA as a percentage of total fatty acids. FLQ is more suitable for seafood due to its high EPA and DHA content. In this study, all the extraction methods showed significant differences in terms of calculated FLQ (*p* < .05). However, previously reported FLQ value ranged from 13.01 to 36.37 for various fish species (Chen & Liu, 2020).

The ratio of LA/ALA is often used to reflect the quality of fats or oils. It was also developed to guide infant formula (Chen & Liu, 2020). However, in the present study, all the extraction methods except SE showed nonsignificant differences in terms of the calculated LA/ALA ratio (*p* > .05). However, obtained values of LA/ALA indices are much higher than the values in milk fat (2.464 ± 0.147) as reported by Sharma et al. (2018).

## 4 CONCLUSIONS

With the aim of achieving premium quality fish oil using different extraction methods, MAE demonstrated to be the highly efficient method for the recovery of oil from pangus fish. This method showed the highest extraction yield and resulted to oil with best physical properties, oxidative stability (AV, PV, FFV), contained important fatty acids (higher content of MUFAs, EPA, DPA, and lower content of SFAs) with optimal nutritional quality index (NQI). Since pangus fish oil contains good quality fatty acids (especially PUFAs), present study suggests that lipids of pangus fish could be used directly in human diet or as supplementary edible oils. These nutritional values and NQI may also be applied as for the clinical evidences to explore their potential usage in disease prevention and treatment. Furthermore, employing the MAE technique has the potential to facilitate the efficient and regular extraction of edible fish oil in the food and pharmaceutical industries. As conventional oil extraction method requires the use of solvent and prolonged operating periods, MAE method presents a more cost‐effective alternative which is economically viable due to its less extraction time and higher extraction rate.

## AUTHOR CONTRIBUTIONS

**Nahidur Rahman:** Conceptualization (equal); formal analysis (equal); methodology (equal); writing – original draft (equal). **Shaharior Hashem:** Software (supporting); validation (supporting); visualization (supporting); writing – review and editing (supporting). **Shireen Akther:** Writing – review and editing (supporting). **Jakia Sultana Jothi:** Conceptualization (lead); formal analysis (lead); funding acquisition (lead); methodology (lead); supervision (lead); validation (lead); visualization (lead); writing – review and editing (lead).

## CONFLICT OF INTEREST STATEMENT

There is no conflict of interest.

## ETHICS STATEMENT

There were no human subject experiments in our study.

## Data Availability

The data that support the findings of this study are available from the corresponding author, upon reasonable request.
